# Editorial: Novel and alternative therapeutic agents for controlling infectious diseases of poultry

**DOI:** 10.3389/fvets.2023.1232983

**Published:** 2023-07-07

**Authors:** Asghar Abbas, Rao Zahid Abbas, Muhammad Asif Raza, Tauseef ur Rehman, Muhammad Azeem Saeed

**Affiliations:** ^1^Department of Pathobiology, Faculty of Veterinary and Animal Sciences, Muhammad Nawaz Shareef University of Agriculture Multan, Multan, Pakistan; ^2^Department of Parasitology, Faculty of Veterinary and Animal Sciences, University of Agriculture Faisalabad, Faisalabad, Pakistan; ^3^Department of Parasitology, The Islamia University of Bahawalpur, Bahawalpur, Pakistan; ^4^School of Medicine, Section of Immunology, Washington University, Washington, DC, United States

**Keywords:** infectious diseases, poultry, prevention, alternative, control

The poultry sector is a robust, quickly developing, and significant business sector globally, addressing one of the biggest farming-based challenges of creating economies and essentially contributing to national Gross Domestic Product (GDP). It also has a significant role in food security and in the reduction of poverty and destitution (1). Nonetheless, its development is hampered by the spread of diseases (viral, bacterial, and parasitic) with huge morbidity and mortality and far and wide dissemination, which have serious financial ramifications for the poultry business (Abbas et al.).

Heightened chicken stock density and population progression result in greater transmission of diseases between birds and humans (2, 3). In addition, the effectiveness of vaccination is limited by the strains of infectious pathogens (4). Furthermore, the sector also sees the development of diseases due to the use of synthetic antibiotics and drugs in poultry feed; consequently, synthetic antibiotics have been completely outlawed in European nations (5).

As a result, the investigation of novel and novel treatment options for the prevention of infectious diseases has remained a fascinating strategy (6, 7). Nanoparticles and plant-based solutions have shown potential effectiveness against viral infections. Chitosan, nanoparticles, and propolis, a plant-inferred item, were viewed as inventive and were successfully used in antiviral drugs to protect against the Newcastle disease septicity (Alkhalefa et al.). Essential oils and botanical substances have also demonstrated noteworthy effectiveness against avian infectious diseases (8).

*Trachyspermum ammi* (Ajwain) has been shown to be a useful ingredient in poultry feed and in a treatment that combines novel plant and probiotics, showing that it can serve as a viable alternative to antibiotics. The antioxidant and anti-inflammatory properties of ajwain make it an effective diet plant for treating salmonellosis (Haq et al.).

In general, this Research Topic provides a concise summary of the current state of research in the area of novel compounds and advancements in poultry infectious disease control. We hope that the research presented in this RT increases our comprehension and offers novel strategies for controlling avian infectious diseases.

## Author contributions

The initial draft of the manuscript was written by AA. RZA and MAR made changes. TuR added additional text. MAS checked the editorial for errors. All authors contributed to the article and approved the submitted version.
